# Miscellaneous Notices

**Published:** 1841-09

**Authors:** 


### Miscellaneous Notices.

*Omission.*—In noticing the proceedings of the last meeting of the American Society of Dental Surgeons, we inadvertently omitted to mention the names of Charles Bromley, of Southampton, England, Mr. Rogers, of London, and Richard Helsby, of Manchester, England, who were elected honorary members.

---

*Opening Address.*—The opening address delivered by Dr. Hayden, at the late meeting of the American Society of Dental Surgeons, held in Philadelphia, we feel assured, will be read with pleasure by every member of the profession. It is not only an able and well written paper, but is highly interesting. It contains the fullest and most accurate history of the origin and progress of dental science in the United States that has ever been given. Having been actively and constantly engaged in the practice of dental surgery for about forty-five years, Dr. Hayden's opportunities for acquiring this knowledge has been ample, and we are gratified that he embraced the occasion of the delivery of this address, to impart it to those of his professional brethren, not so conversant with the early history of the science in this country. With its rapid advance during the last fifteen or twenty years, all are more or less familiar, but with its introduction into, and its early progress in the United States, few comparatively, were before acquainted.

The name of Greenwood, it is true, is known in almost every part of the civilized world, and though one among the first to exercise the duties of the dentist in this country, he ranked high in the profession during his time, but there were others who preceded, and that were cotemporary with him, and who, though not so extensively known, were nevertheless, possessed of much merit. Dr. H. has, therefore, conferred a favour in thus rescuing their names from forgetfulness, and placing them among those of the early cultivators of the art.

---

*Preservation of the Teeth.*—We earnestly invite the attention of the profession to the dissertation, read by Dr. L. S. Parmly, before the American Society of Dental Surgeons, at their last meeting, on the "Management of the Mouth and Teeth." If the truths that are therein so ably and forcibly urged, were more strongly impressed on the minds of dental practitioners, than they seem generally to be, the amount of their usefulness would be greatly increased, and much of the evil, resulting from the destruction of the teeth, which they are daily called upon to remedy, might be prevented. We hope, therefore, that more attention than has hitherto been bestowed, will be given to the prevention of disease in these organs, and that every dental surgeon, will not only urge its importance upon his patients, and more especially upon the heads of families in which he practises, but will also supply them with the means recommended by Dr. Parmly, for its accomplishment. With floss silk, argillaceous tooth polishers, and tongue scrapers, every practitioner should be provided, and if they cannot be procured elsewhere, they may be obtained from the New York editor of this Journal, Dr. Solyman Brown, 13 Park Place, who keeps a large and constant supply. As for ourself, we should feel that we were doing great injustice to our patients, were we not to keep them, and recommend their use.—*Balt. Ed.*

---

*Professor Baumes' Work.*—In giving place to the transactions of the "American Society of Dental Surgeons," we are compelled to occupy the library portion of this number of our Journal. We shall endeavour, however, to make up for this, by giving nearly the whole of our next to the continuation of Dr. Bond's translation of Professor Baumes' excellent treatise on first dentition. In pursuing this course, we are under the necessity of delaying the publication of several articles now on hand. We hope, therefore, we shall be excused by our correspondents for what might otherwise seem intentional neglect.

---

*Materials proper for Plugging Teeth.*—In a letter which will be found in another part of our Journal, addressed to the American Society of Dental Surgeons, by Dr. J. H. Foster, of New York, the superiority of gold over all other articles or materials employed for stopping teeth, is clearly and satisfactorily set forth. There can be but one opinion among well informed practitioners on this subject. All agree, that while there are many cases in which tin may be advantageously employed, pure gold is the only metal or material, except platina, that can in all cases be relied upon. It is incomparably better than any other substance. We commend to our readers a careful perusal of Dr. F's letter.

---

*Popular Essays on Dental Surgery.*—Six or seven of the essays ordered by the American Society of Dental Surgeons, have been published, and every member whose dues have been paid is entitled to twenty-five copies of each, gratis, but for all over that number, the rate of one dollar and fifty cents per one hundred, will be charged. We would advise the members of the Society generally to supply themselves with them. Much good may be accomplished by their distribution, inasmuch as they are calculated to do away with the false opinions which many entertain concerning this branch of the medical art, by the dissemination of correct views upon the subject. We think, therefore, that every well-informed member of the profession should contribute to the accomplishment of an object so much to be desired.

---

*Annual Dues.*—To carry into effect the objects contemplated by the American Society of Dental Surgeons, and among which, are the publication and distribution of the essays alluded to in the preceding notice, also, the publication of such other papers as may be contributed to it, tending to the advancement of dental science, and the procurement of a library, it is necessary that every member should be punctual and prompt in the payment of his annual dues. Besides, no member is entitled to vote on any question at the meetings of the society, until he shall have paid his membership fee, five dollars. Such of the members then as have not complied with this requisition of the by-laws of the society, we hope will lose no time in doing it. Dr. E. Baker, No. 6 Warren street, N. Y., is the treasurer, and the proper person to receive and receipt for all moneys paid to the society.

---

*Mr. Charles Brown, Surgeon Dentist, of Woolwich, Kent, England.*—The Baltimore editor takes this opportunity of returning to the gentleman whose name heads this notice, his most grateful acknowledgments for the beautiful drawing of the microscopic view of the internal membrance of a superior bicuspis, and for the letter which accompanied it—both of which is copied into the present number of the Journal. For the kind interest which Mr. B. manifests in behalf of the efforts that are at this time being made in the United States, for the elevation and advancement of dental science, he also feels profoundly grateful, and rejoices to learn that his professional brethren on the other side of the water, are disposed to lend a helping hand to a work, which, if *zealously* and *resolutely* prosecuted, must soon eventuate in placing the calling of the surgeon dentist on a footing with that of the other branches of medicine. This is a consummation earnestly to be desired, and it is to be hoped, that the period is not distant when it will be accomplished.

*Embellishments.*—The two plates with which this number of our Journal is enriched, were lithographed by Messrs. E. Weber & Co. of Baltimore, than whom, in this useful and most beautiful art, none, perhaps, in this country have attained to greater skill or perfection. The likeness of Doctor Hayden was taken from a painting executed sixteen years ago, but notwithstanding the effects of time, no one acquainted with the Doctor can fail to recognize in this, a strong resemblance to the original. The expression of the eyes and whole countenance, and the contour of the face and head are perfect.

The coloured plate is worthy of especial notice, as a fine specimen of a new and highly useful improvement in lithography. Until very recently, colouring was done by the pencil, and of course, was a very tedious and expensive process. Hence, anatomical plates, &c. have been so costly as to be beyond the reach of many who desired to have them. The art of colouring in lithography, we believe, was introduced into this country by Messrs. Weber & Co. and of the accuracy and beauty of which, our readers have an opportunity of juding from the specimen furnished in this number of our Journal. It is needless, therefore, to commend these artists to those who may have occasion to employ lithographers. Their work will do this more effectually than any thing which we can say upon the subject.

---

*Singular Phenomenon.*—We were presented a few months since, by Dr. Jenks, Dentist, of Fredericktown, Md., with a superior molar tooth, on one of the sides of the neck of which, about the eighth of an inch above the termination of the enamel upon the crown, and just where the bifurcation of two of its roots takes place, is a protuberance, the size of a pin's head, or perhaps, a little larger, covered with enamel. Now, what seems remarkable in this, is, that this protuberance is covered with enamel. We can very readily conceive that it could have been formed by a deposition of bone, but the presence of the enamel, according to the prevailing theory of the manner of the formation of this substance, renders its explanation somewhat difficult.

Since writing the above, another molar tooth, having a similar enamelled wart-like protuberance upon it, was presented to us by Mr. Savier, student of Dental Surgery, of Baltimore.—*Balt. Ed.*

---

*Progress of Dental Science in America.*—Under this head a writer in the London Lancet speaks very favourably of the praiseworthy exertions recently made in this country by some of our leading surgeon-dentists. It is well known that these exertions have resulted in the organization of the "Society of Dental Surgeons," and the commencement of the "Journal of Dental Science," both of which are justly extolled by the writer alluded to.

[*Boston Med. and Surg. Journal.*

